# Association between antibiotic exposure and survival outcomes in hepatocellular carcinoma patients treated with immune checkpoint inhibitors: a systematic review and meta-analysis

**DOI:** 10.7717/peerj.21840

**Published:** 2026-10-28

**Authors:** Shuaihang Chen, Shanshan Chen, Yuanqiang Li, Jimin Su, Chengze Yu, Congxi Xu, Qinglin Li, Qibiao Wu, Changgang Sun

**Affiliations:** 1 Faculty of Chinese Medicine, Macau University of Science and Technology, Macau, China; 2 The First Clinical Medical College, Zhejiang Chinese Medical University, Hangzhou, China; 3 Tongde Hospital of Zhejiang Province Affiliated to Zhejiang Chinese Medical University (Tongde Hospital of Zhejiang Province), Hangzhou, China; 4 Postgraduate Training Base Alliance of Wenzhou Medical University (Zhejiang Cancer Hospital), Hangzhou, China; 5 Clinical Medical College of Integrated Traditional Chinese and Western Medicine of Zhejiang Chinese Medical University, Hangzhou, China; 6 Weifang Traditional Chinese Hospital, Weifang, China

**Keywords:** Immune checkpoint inhibitors, Hepatocellular carcinoma, Antibiotic, Overall survival, Progression-free survival

## Abstract

**Objective:**

Patients with hepatocellular carcinoma (HCC) treated with immune checkpoint inhibitors (ICIs) are frequently exposed to antibiotics. This study aims to evaluate the impact of antibiotic use on the survival outcomes of HCC patients treated with ICIs.

**Methods:**

This study followed the PRISMA guidelines and systematically retrieved literature from PubMed, Embase, and Cochrane Library databases up to August 7, 2026. Studies comparing the clinical outcomes (overall survival (OS), progression-free survival (PFS), objective response rate (ORR), and disease control rate (DCR)) of HCC patients receiving ICI treatment with and without antibiotic exposure were included. The quality of the studies was assessed using the Newcastle-Ottawa Scale. Before conducting the meta-analysis, the original hazard ratios (HRs)/odd ratios (ORs) and their 95% CIs were log-transformed to achieve a normal distribution. Statistical analysis was performed using Stata 15 software, and pooled HRs/ORs with 95% CIs were calculated.

**Results:**

A total of 12 retrospective cohort studies were included, involving 3,643 patients (761 exposed to antibiotics). The results showed that the median OS of the antibiotic-exposed group (12.2 months) was shorter than that of the non-exposed group (17.0 months). The meta-analysis indicated that antibiotic use was significantly associated with poorer OS (HR = 1.29, 95% CI [1.17–1.41]). Sensitivity analyses confirmed the robustness of the OS finding. In terms of PFS, the median PFS of the antibiotic-exposed group (5.2 months) was shorter than that of the non-exposed group (7.5 months), and antibiotic use was also significantly associated with poorer PFS (HR = 1.27, 95% CI [1.01–1.59]), although this association was not robust in sensitivity analyses. The analyses of ORR and DCR did not show significant differences between the groups. No significant publication bias was detected on the basis of visual inspection of funnel plots and Egger’s and Begg’s tests.

**Conclusion:**

For HCC patients receiving ICI therapy, antibiotic exposure was significantly associated with reduced OS. A significant association with poorer PFS was also observed, although this finding was not robust in sensitivity analyses and should therefore be interpreted with caution. No statistically significant differences were observed for ORR or DCR.

## Introduction

Hepatocellular carcinoma (HCC) is the most common primary liver cancer and represents a substantial and persistent challenge to global healthcare systems (Vogel et al., 2022). Owing to the difficulties in early detection and diagnosis, the five-year survival rate of HCC remains as low as 18%, rendering it the third leading cause of cancer-related mortality worldwide (Sung et al., 2021; Siegel et al., 2022). In the setting of advanced disease, immune checkpoint inhibitors (ICIs) have emerged as a cornerstone therapeutic option (Yang et al., 2024; Sensi et al., 2024). However, the clinical benefit of ICIs is far from universal. In clinical practice, over 70% of patients eventually develop resistance to these agents (Qin et al., 2023; Cui et al., 2025). The determinants of patient response to ICIs are multifactorial and remain incompletely understood. A growing body of evidence has implicated a diverse array of molecular features, including tumor-associated macrophage polarization, MAPK pathway activation, epigenetic modifications, non-coding RNA regulatory networks, and copper metabolism-related pathways (Liu et al., 2025a; Ribas et al., 2020; Tao et al., 2023; Shao et al., 2023; Zhu et al., 2024b; Shen et al., 2020).

Intriguingly, the gut microbiota has recently garnered attention as a potential modulator of ICI responses (Gunjur et al., 2024). Accumulating evidence suggests that the efficacy of these immunotherapies across various cancers is linked to the composition and abundance of a patient’s gut bacterial community (Derosa et al., 2022; McCulloch et al., 2022; Mao et al., 2021). This observation poses a clinical conundrum: antibiotics—frequently prescribed to patients with advanced cancers—are known to disrupt the delicate gut ecosystem, triggering a decline in microbial diversity and impairing key metabolic functions within the microbiome (Fishbein, Mahmud & Dantas, 2023; Schell & Carmody, 2025; Anthony et al., 2022). Mounting clinical evidence now implicates this disruption as a possible contributor to diminished ICI effectiveness. Indeed, analyses across multiple cancer types, including non-small cell lung cancer, melanoma, and renal cell carcinoma, have consistently demonstrated that antibiotic exposure around the time of ICI treatment correlates with poorer survival outcomes, such as reduced overall survival (OS) or progression-free survival (PFS) (Tsikala-Vafea et al., 2021; Zhou et al., 2022; Abdelhamid et al., 2023; Gambichler et al., 2025).

Nevertheless, the specific impact of antibiotic use on ICI outcomes in hepatocellular carcinoma remains far from settled, with recent studies yielding notably mixed—and at times contradictory—survival data. For instance, Fessas et al. (2021) counterintuitively reported significantly better progression-free survival among patients who had received antibiotics. Conversely, Wang et al. (2022) found no significant association between antibiotic exposure and survival outcomes, whereas Cheung et al. (2021) observed the opposite pattern: higher mortality and poorer survival in the antibiotic-exposed group. Given these starkly discrepant findings and their far-reaching implications for clinical practice, we conducted a comprehensive systematic review and meta-analysis to reconcile the existing evidence and provide a more definitive, pooled assessment.

## Methods

### Study design and registration

This systematic review and meta-analysis was conducted in accordance with the PRISMA guidelines (see File S1) (Page et al., 2021). To ensure transparency and minimize reporting bias, a detailed study protocol was prospectively developed and registered on the PROSPERO international registry (ID: CRD420251248950).

### Search strategy

A comprehensive literature search was carried out by two independent investigators (CSH and CSS), under the supervision of senior researchers (SCG, LQL and WQB). We systematically queried three major electronic databases—PubMed, Embase, and the Cochrane Library—for relevant records published from inception up to August 7, 2026. To balance sensitivity and precision, the search strategy incorporated both controlled vocabulary (*e.g.*, Medical Subject Headings (MeSH)) and free-text keywords. The full search syntax for each database is provided in File S2. Furthermore, to minimize the risk of omitting eligible studies, we manually examined the reference lists of key review articles and other relevant publications. All searches were restricted to articles published in English. Should critical data be missing from any included study, we planned to contact the corresponding authors *via* email to request the necessary information.

### Study selection

The screening of titles, abstracts, and full texts was performed independently by the same two investigators (CSH and CSS) using a blind parallel procedure. Any disagreements regarding a study’s eligibility were resolved through discussion, with arbitration by a third reviewer (SCG) if consensus could not be reached. For studies where methodology or outcomes were ambiguously reported, we contacted the corresponding authors by email to seek clarification before finalizing inclusion.

#### Inclusion criteria

1. Adult patients (≥18 years) with a confirmed HCC diagnosis;
2. Comparison between an antibiotic-exposed cohort and a non-exposed control cohort;
3. Reporting of survival outcomes (*e.g.*, PFS or OS);
4. Case-control or cohort study design.

#### Exclusion criteria

1. Duplicate reports;
2. Non-peer-reviewed study (*e.g.*, abstracts, preprint);
3. Lack of reported survival data and unavailability of data upon author contact;
4. Non-English language.

### Quality assessment and data extraction

Two reviewers (CSH and CSS) conducted independent quality assessments using the Newcastle-Ottawa Scale (NOS) (Stang, 2010). Disagreements were adjudicated by a third researcher (WQB).

Two reviewers (CSH and CSS) independently extracted data, which covered: (1) study identifiers and design (author, year, country, study type, duration); (2) HCC tumor stage; (3) exposure details (reason for antibiotic use, antibiotic class, ICI type, timing of antibiotic exposure); (4) covariates from multivariable models for OS/PFS; (5) key outcomes (PFS, OS, Objective Response Rate (ORR), Disease Control Rate (DCR)). We used WebPlotDigitizer 4.5 to digitize data from the survival curves and calculated the hazard ratios (HRs) with 95% confidence intervals (CIs) using the method of (Tierney, Burdett & Fisher, 2025). For survival outcomes (OS and PFS), we preferentially extracted multivariable-adjusted HRs with 95% CIs. For binary outcomes (ORR and DCR), we preferentially extracted multivariable-adjusted odds ratios (ORs).

### Statistical analysis

All statistical analyses were performed using Stata 15 (StataCorp LLC, College Station, TX, USA). Since the HR may be skewed, before conducting the meta-analysis, we performed a natural logarithm transformation on the original HRs/ORs and their 95% CIs in each study to achieve a normal distribution. The association between antibiotic use and ICI treatment efficacy was reported as ORs with 95% CIs. The impact of antibiotic use on survival risk in HCC patients was quantified using HRs and 95% CIs. We quantified between-study heterogeneity with the *I*^2^ statistic and Cochran’s test: a fixed-effects model was used for *I*^2^ ≤ 50% (*p* > 0.05), and a random-effects model otherwise (Schmidt, Oh & Hayes, 2009). Result robustness was tested with sensitivity analyses. Publication bias was assessed using Egger’s test, Begg’s test, and funnel plots (Egger et al., 1997).

## Results

### Study selection

The study selection flow is presented in Fig. 1. The database searches retrieved 876 publications. Following the removal of 75 duplicates, 801 unique records were screened, resulting in the inclusion of 12 retrospective cohort studies (Fessas et al., 2021; Wang et al., 2022; Cheung et al., 2021; Ng et al., 2025; Li et al., 2025; Pinato et al., 2023; Maesaka et al., 2023; Alshammari et al., 2023; Hatanaka et al., 2023; Spahn et al., 2020; Ren et al., 2026; Yi et al., 2025). These studies, conducted across six countries/regions (including a multi-regional study by Fessas et al. (2021)), enrolled 761 patients with antibiotic exposure and 2,882 unexposed patients. All studies reported relevant survival outcomes. Effect estimates were derived from confounder-adjusted analyses in all studies except (Spahn et al., 2020). Most studies employed multivariable Cox models, with (Ren et al., 2026) instead using a 1:1 propensity score-matched analysis. Detailed baseline characteristics are provided in Table 1. Further details on the types of ICIs and antibiotic usage can be found in File S3.

**Figure 1:**
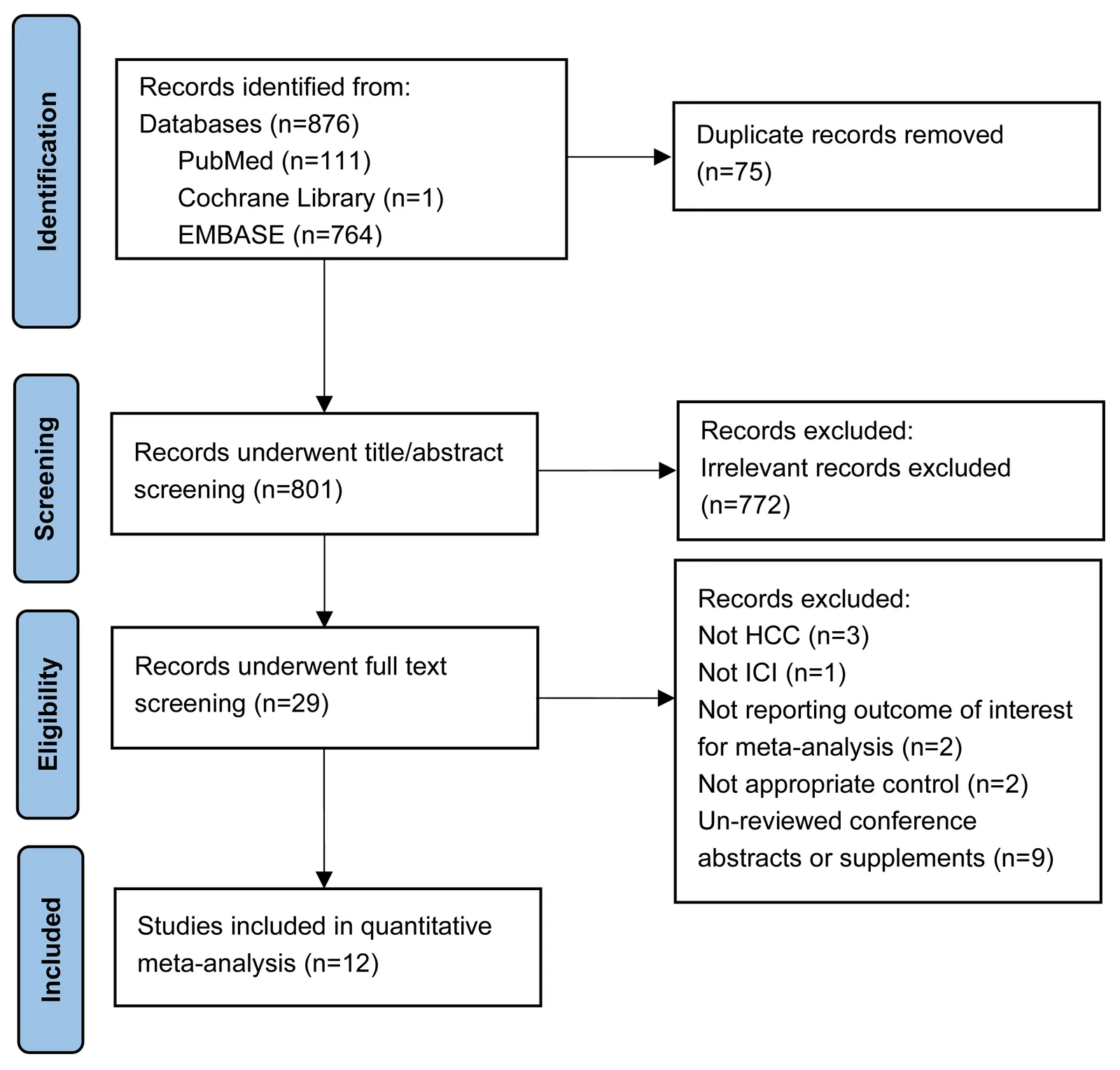
PRISMA diagram describing the process of study selection. HCC, hepatocellular carcinoma; ICI, immune checkpoint inhibitors.

**Table 1:** Characteristics of the included studies.

Author, year	Country	Study duration	HCC stage	Sample size		Immunotherapy	Outcomes	Covariates of multivariable analysis
				ATB exposure	Non-ATB exposure			
Ren et al. (2026)	China	From January 1, 2019, to December 31, 2024	advanced HCC	20	20	PD-1 (camrelizumab, sintilimab, tislelizumab)	OS, PFS	PSM matching: age, sex, treatment modality, alcohol use, Child-Pugh class, BCLC stage, HBV DNA level, antiviral status, ALT, AST, GGT, ALP, TBIL, ALB, PT, WBC, PLT, AFP.
Yi et al. (2025)	China	From January 2018 to December 2022	Unresectable HCC	78	773	PD-1/PD-L1(pembrolizumab, tislelizumab, sintilimab, atezolizumab, durvalumab)	OS, PFS	Age, sex, etiology, ECOG PS, Child-Pugh class, BCLC stage, AFP, tumor diameter, tumor number, macrovascular invasion, extrahepatic spread, NLR, immunotherapy regimen, treatment line.
Ng et al. (2025)	Singapore	From May 2015 to November 2019	advanced HCC	62	106	–	OS, PFS, ORR, DCR	ECOG PS, etiology, Child-Pugh score, AFP, as well as predetermined ones such as ICI treatment.
Li et al. (2025)	China	From January 1, 2021 to December 30, 2023	advanced HCC	73	29	–	OS, PFS	OS: Sex. PFS: AFP.
Pinato et al. (2023)	America	From 2016 to 2019	unresectable/metastatic HCC	120	660	PD-1/PD-L1 inhibitors, CTLA-4 antagonists	OS, PFS	Age, race, sex, region, ECOG PS, aetiology of chronic liver disease, presence of macrovascular invasion, presence of extrahepatic disease, and receipt of prior lines of treatment.
Maesaka et al. (2023)	Japan	From October 2020 to January 2022	unresectable HCC	19	86	PD-L1 inhibitors (atezolizumab)	OS, PFS	OS: Child-Pugh score; Maximum intrahepatic tumor size; Intrahepatic tumor number; Macrovascular invasion; AFP, NLR, CRP. PFS: Intrahepatic tumor number, Macrovascular invasion, NLR, CRP.
Alshammari et al. (2023)	Saudi Arabia	–	advanced HCC	20	39	PD-L1 inhibitors (Nivolumab)	OS	Gender, Age, Diabetes mellitus, Hypertension, Cardiac disease, Chronic kidney disease, Presence of cirrhosis.
Hatanaka et al. (2023)	Japan	From September 2020 to April 2022	–	43	384	PD-L1 inhibitors (atezolizumab)	OS, PFS	Age, sex, viral infection, BCLC stage, Child-Pugh score, ALBI score, treatment settings, serum albumin, total bilirubin, FIB-4 score, serum AFP, and serum DCP.
Wang et al. (2022)	China	From June 2018 to October 2020	–	41	174	PD-1 inhibitors	OS, PFS, DCR	OS: tumor thrombus, Child-Pugh, AFP. PFS: Extrahepatic metastasis, AFP. DCR: Extrahepatic metastasis, Subsequent therapy, AFP
Fessas et al. (2021)	Europe, the USA, Asia	From 2017 to 2019	–	155	247	PD-1/PD-L1 inhibitors, CTLA-4 antagonists	OS, PFS, ORR, DCR	Gender, age, viral etiology, Child-Turcotte-Pugh class, BCLC stage, ICI treatment, treatment line, and AFP
Cheung et al. (2021)	China	From January 2014 to December 2019	advanced HCC	109	286	PD-1 inhibitors, CTLA-4 antagonists (nivolumab, pembrolizumab, ipilimumab)	OS	Age, sex, type of immunotherapy, prior hepatectomy/TACE/ablative therapy, alcohol-related diseases, DM, hypertension, IHD, AF, CHF stroke, hepatic decompensation, cause of cirrhosis, prednisolone, aspirin, statins, PPIs, prior sorafenib/lenvatinib use, AFP, bilirubin, ALP, ALT, AST, albumin, globulin, INR, platelet, creatinine, sodium.
Spahn et al. (2020)	Europe	From August 2015 to December 2019	advanced HCC	21	78	PD-L1 inhibitors (nivolumab, pembrolizumab)	OS, PFS	Not reported

**Notes.**

HCC hepatocellular carcinoma PSM propensity score-matched ATB antibiotic PD-1 programmed cell death protein 1 PD-L1 programmed cell death ligand 1 CTLA-4 cytotoxic T-lymphocyte-associated protein 4 OS overall survival PFS progression-free survival ORR objective response rate DCR disease control rate ICI immune checkpoint inhibitor ECOG PS Eastern Cooperative Oncology Group performance status BCLC Barcelona Clinic Liver Cancer Child-Pugh Child-Pugh class ALBI albumin-bilirubin NLR neutrophil-to-lymphocyte ratio FIB-4 fibrosis index based on four factors AFP alpha-fetoprotein HBV hepatitis B virus ALT alanine aminotransferase AST aspartate aminotransferase GGT gamma-glutamyl transferase ALP alkaline phosphatase TBIL total bilirubin ALB albumin PT prothrombin time WBC white blood cell count PLT platelet count INR international normalized ratio TACE transarterial chemoembolization PPIs proton pump inhibitors CRP C-reactive protein DCP des-gamma-carboxy prothrombin AF atrial fibrillation CHF congestive heart failure DM diabetes mellitus IHD ischemic heart disease

### Quality assessment

The methodological quality of all included studies was assessed using the NOS; detailed scoring is presented in File S4. The evaluation yielded a mean score of 8.17 across the 12 studies. Points were primarily lost for inadequate control of confounding factors and insufficient or unreported follow-up duration. All studies attained an NOS score of 7 or higher. The consistently high scores indicate that the included studies had no major design flaws, supporting the reliability of their findings.

### Overall survival

Ten studies (Fessas et al., 2021; Ng et al., 2025; Li et al., 2025; Pinato et al., 2023; Maesaka et al., 2023; Alshammari et al., 2023; Hatanaka et al., 2023; Spahn et al., 2020; Ren et al., 2026; Yi et al., 2025) reported median OS. The antibiotic-exposed (ATB+) group had a pooled median OS of 12.2 months (range: 5.0–18.2), which was shorter than the 17.0 months (range: 10.0–24.0) observed in the non-antibiotic-exposed (ATB-) group (two studies did not reach median OS by 15-month follow-up; Fig. 2A). In all ten studies, the median OS was longer in the ATB- group than in the ATB+ group. A meta-analysis of OS was conducted using data from 12 studies (Fessas et al., 2021; Wang et al., 2022; Cheung et al., 2021; Ng et al., 2025; Li et al., 2025; Pinato et al., 2023; Maesaka et al., 2023; Alshammari et al., 2023; Hatanaka et al., 2023; Spahn et al., 2020; Ren et al., 2026; Yi et al., 2025) (*n* = 3,643 patients: 761 ATB+ and 2,882 ATB-). Due to the absence of substantial heterogeneity (*I*^2^ = 6.2%, *P* = 0.384), a fixed-effects model was used. The combined HR estimates showed that the antibiotic exposure was significantly associated with worse OS (HR = 1.29, 95% CI [1.17–1.41]) in HCC patients receiving ICI therapy (Fig. 2B).

**Figure 2:**
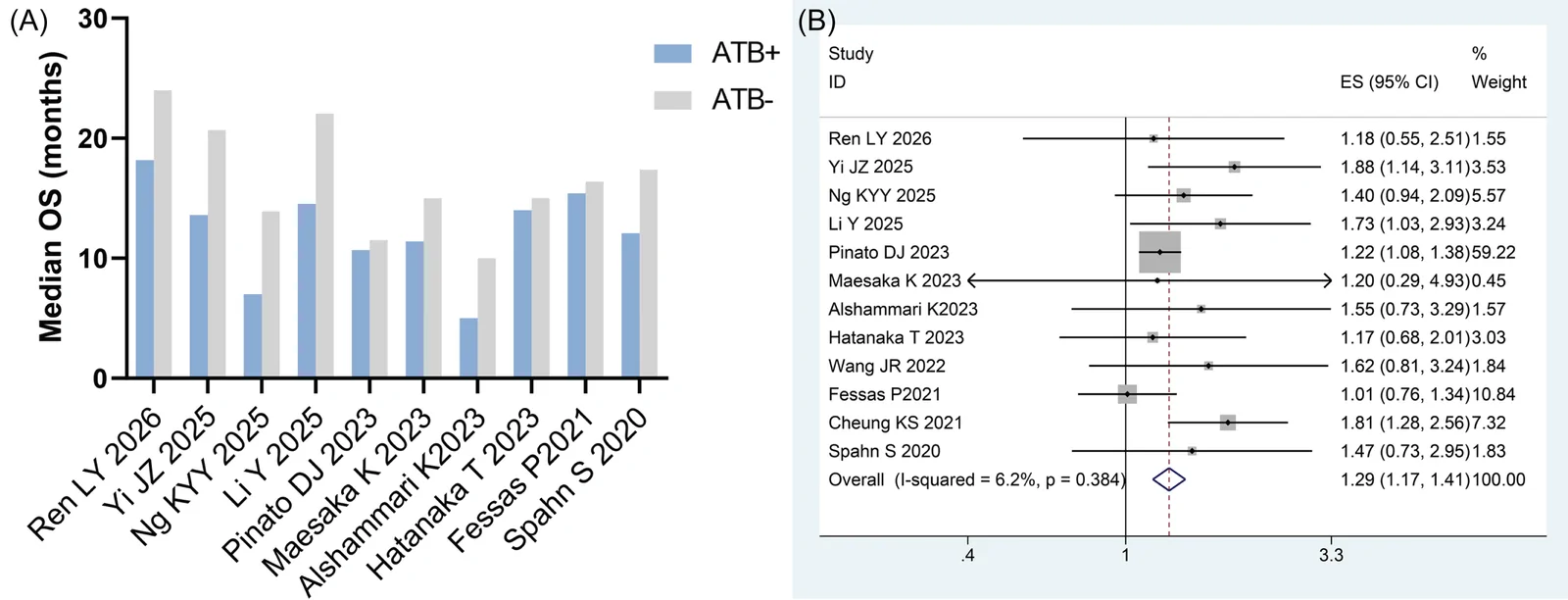
(A) Median OS of each study. (B) Meta-analysis of the overall OS. OS, overall survival; HR, hazard ratio; CI, confidence interval.

### Progression-free survival

Ten studies (Fessas et al., 2021; Wang et al., 2022; Ng et al., 2025; Li et al., 2025; Pinato et al., 2023; Maesaka et al., 2023; Hatanaka et al., 2023; Spahn et al., 2020; Ren et al., 2026; Yi et al., 2025) reported median PFS. The median PFS was shorter in the ATB+ group (5.2 months; range: 2.1–7.6) than in the ATB- group (7.5 months; range: 2.4–12.3) (Fig. 3A). With the exception of the study by Fessas et al. (2021) (which reported superior PFS in the ATB+ group), all others indicated poorer PFS outcomes associated with antibiotic exposure. The PFS meta-analysis pooled data from these 10 studies (Fessas et al., 2021; Wang et al., 2022; Ng et al., 2025; Li et al., 2025; Pinato et al., 2023; Maesaka et al., 2023; Hatanaka et al., 2023; Spahn et al., 2020; Ren et al., 2026; Yi et al., 2025), encompassing 3,189 patients (632 ATB+ and 2,557 ATB-). Given substantial heterogeneity (*I*^2^ = 77.2%, *P* < 0.001), a random-effects model was applied. The pooled HR analysis revealed that antibiotic use was significantly associated with poorer PFS in HCC patients receiving ICI therapy (HR = 1.27, 95% CI [1.01–1.59]; Fig. 3B).

**Figure 3:**
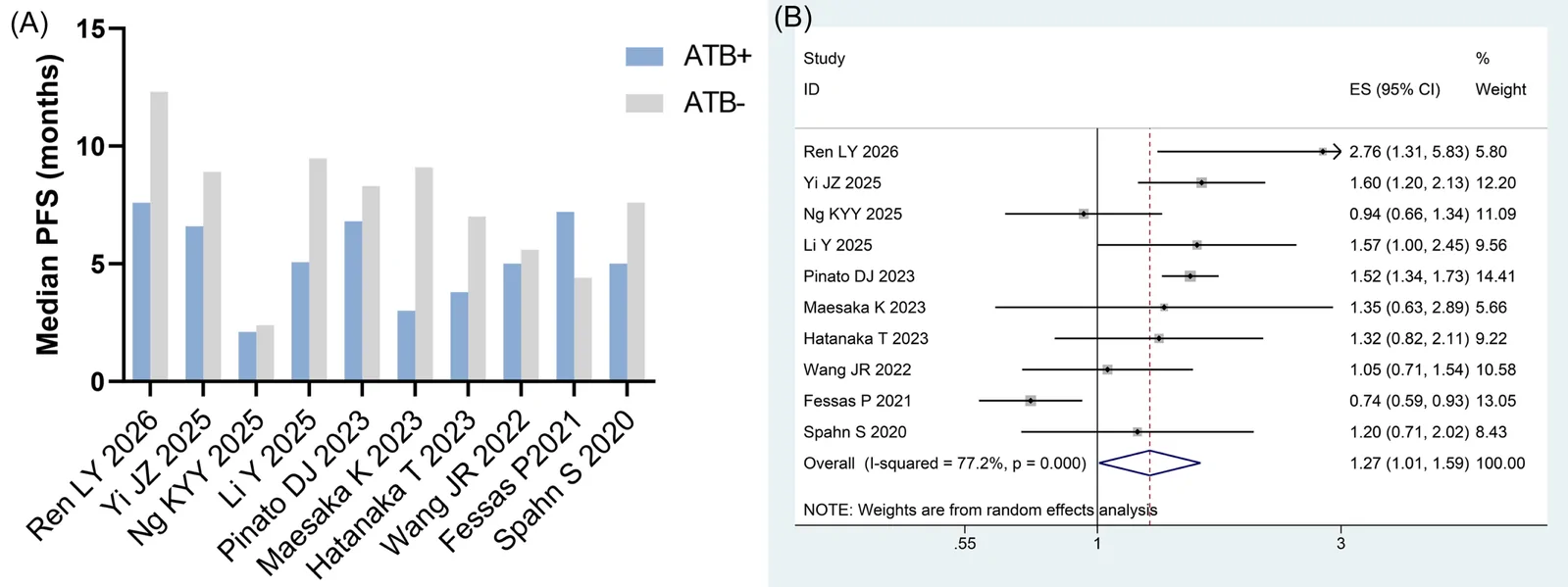
(A) Median PFS of each study. (B) Meta-analysis of the overall PFS. PFS, progression-free survival; HR, hazard ratio; CI, confidence interval.

### Objective response rate and disease control rate

Although several other included studies also reported ORR and DCR outcomes, only two studies (Fessas et al., 2021; Ng et al., 2025) and three studies (Fessas et al., 2021; Wang et al., 2022; Ng et al., 2025) reported ORR and DCR data with extractable odds ratios, and were thus the only studies eligible for pooling in these meta-analyses. Meta-analyses were performed using fixed-effect models given low heterogeneity (ORR: *I*^2^ = 0.0%, *P* = 0.951; DCR: *I*^2^ = 34.7%, *P* = 0.216). The combined results showed that antibiotic use was not significantly associated with ORR or DCR in ICI-treated HCC patients (ORR: OR = 1.30, 95% CI [0.84–2.02]; DCR: OR = 1.20, 95% CI [0.86–1.66]; Figs. 4A and 4B).

**Figure 4:**
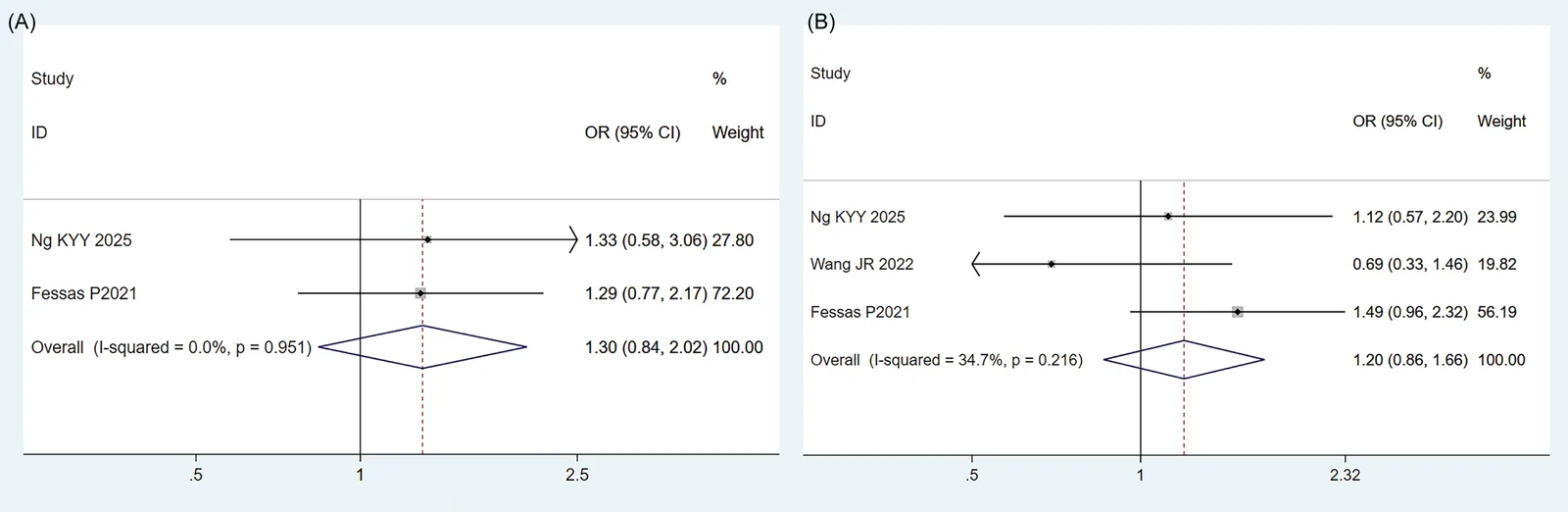
(A) Meta-analysis of the overall ORR. (B) Meta-analysis of the overall DCR. ORR, objective response rate ; DCR, disease control rate; OR, odds ratio; CI, confidence interval.

### Sensitivity analysis and publication bias

Leave-one-out sensitivity analyses were conducted to evaluate the robustness of the primary pooled estimates for OS and PFS (Figs. 5A and 5B). For OS, the pooled estimate remained statistically significant and directionally consistent after exclusion of any single study, supporting the robustness of the OS finding. For PFS, the pooled estimate likewise remained directionally consistent across all leave-one-out iterations; however, statistical significance was lost after the exclusion of seven of the ten studies (Ren et al., 2026; Yi et al., 2025; Li et al., 2025; Pinato et al., 2023; Maesaka et al., 2023; Hatanaka et al., 2023; Spahn et al., 2020), indicating that the PFS association was not robust and should be interpreted with caution. Visual inspection of the funnel plots revealed no overt asymmetry, suggesting a low risk of publication bias (Figs. 6A and 6B). This finding was further corroborated by Egger’s test (OS: *p* = 0.145; PFS: *p* = 0.777) and Begg’s test (OS: *p* = 0.837; PFS: *p* = 0.721), both of which yielded non-significant results, collectively supporting the absence of substantial publication bias (Figs. 7A–7D).

**Figure 5:**
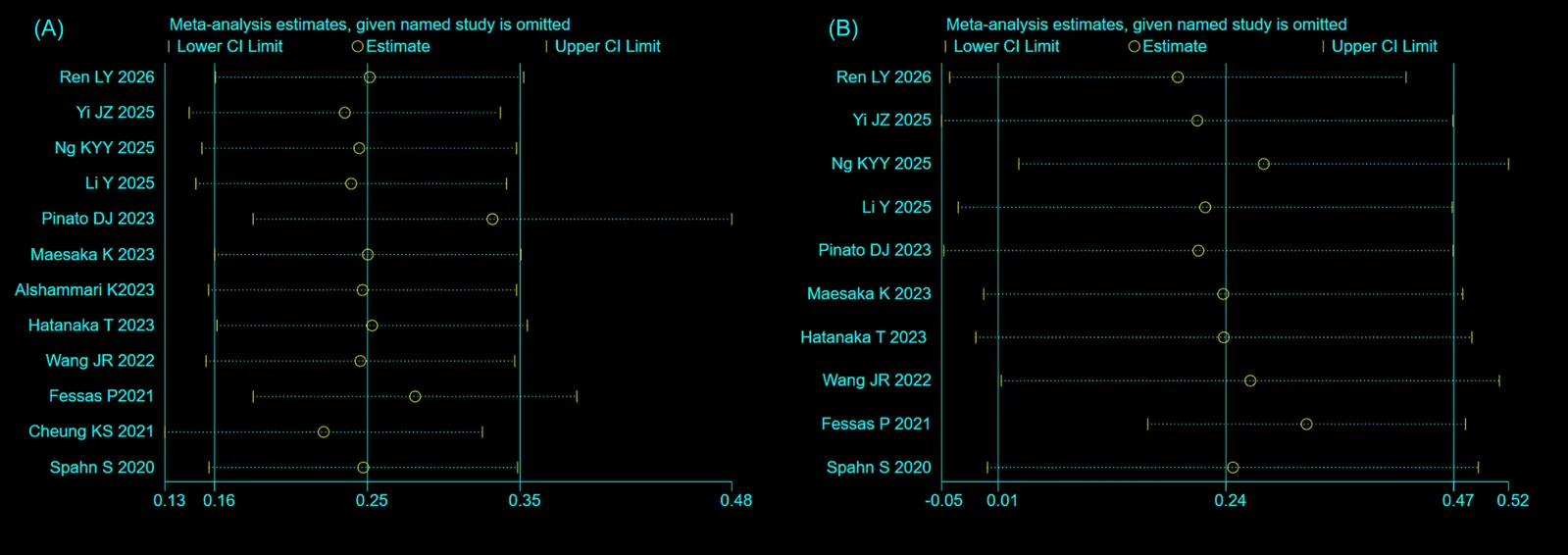
(A) Sensitivity analysis plot of OS. (B) Sensitivity analysis plot of PFS. OS, overall survival; PFS, progression-free survival; CI, confidence interval.

**Figure 6:**
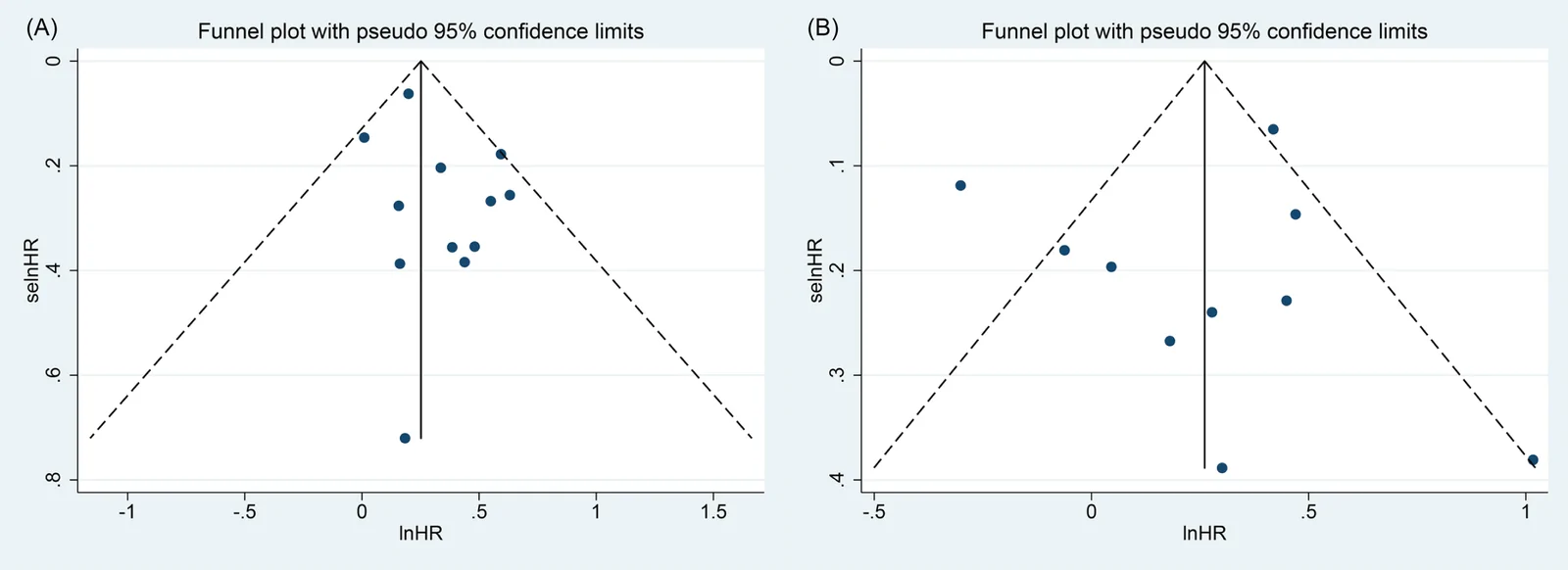
(A) Funnel plot of OS. (B) Funnel plot of PFS. OS, overall survival; PFS, progression-free survival; HR, hazard ratio.

**Figure 7:**
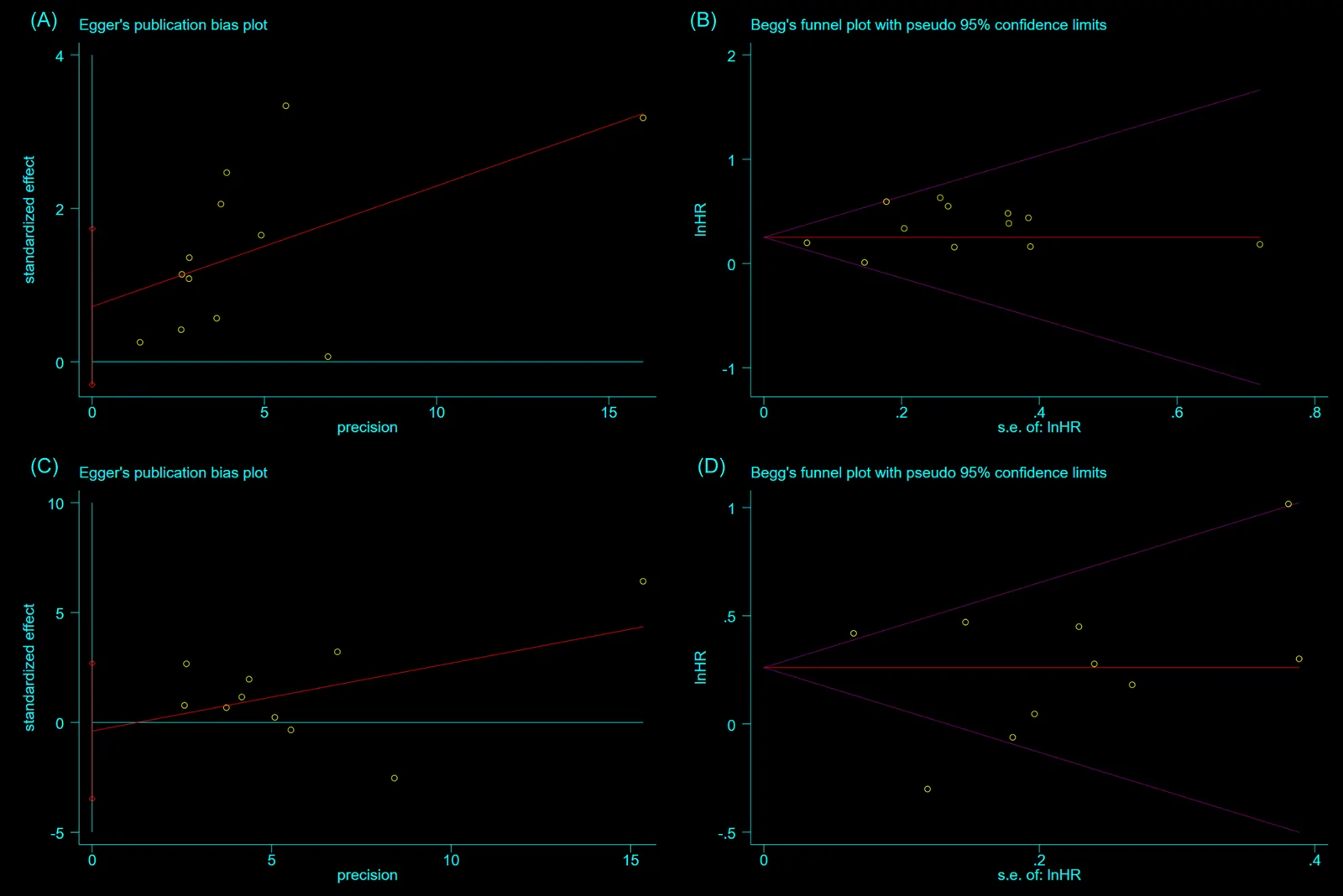
(A) Egger’s publication bias plot of OS, (B) Begg’s publication bias plot of OS, (C) Egger’s publication bias plot of PFS, (D) Begg’s publication bias plot of PFS. OS, overall survival; PFS, progression-free survival; HR, hazard ratio.

## Discussion

In routine clinical practice, antibiotics are frequently administered to patients with HCC for the management of infections, procedural prophylaxis, or empirical treatment. Given that ICIs have been established as a cornerstone therapy for advanced HCC, elucidating the potential impact of concomitant antibiotic use on ICI efficacy has emerged as a pressing clinical question. In 2022, Zhang et al. (2022) published the first meta-analysis addressing this issue, which pooled data from six studies. Notably, their synthesis concluded that—in contrast to observations in other cancer types—antibiotic exposure did not significantly affect survival outcomes in HCC patients receiving ICIs, a finding that was considered noteworthy and somewhat unexpected at the time. However, several additional investigations (Wang et al., 2022; Ng et al., 2025; Li et al., 2025; Pinato et al., 2023; Maesaka et al., 2023; Alshammari et al., 2023; Hatanaka et al., 2023; Ren et al., 2026; Yi et al., 2025) have since been published, necessitating an updated and more comprehensive assessment. The present meta-analysis builds upon and extends this earlier work by adopting stricter inclusion criteria (restricted to peer-reviewed English-language publications), incorporating a larger number of studies, and encompassing a substantially expanded patient cohort, thereby yielding more robust and definitive evidence.

The present updated synthesis comprised 12 high-quality, peer-reviewed studies (all with a NOS score ≥ 7) originating from six countries or regions, encompassing a total of 3,643 patients with HCC (761 antibiotic-exposed and 2,882 antibiotic-unexposed). A key finding was the identification of a significant association between antibiotic use during ICI treatment and poorer OS. The pooled median OS was shorter in the antibiotic-exposed group than in the non-exposed group. The pooled analysis of all 12 studies demonstrated a consistent detrimental effect on OS, with low between-study heterogeneity—a conclusion that diverges from the findings of prior meta-analyses (Zhang et al., 2022). Sensitivity analyses confirmed the robustness of the OS finding, as the pooled estimate remained statistically significant after the exclusion of any single study. With respect to PFS, the pooled median PFS was likewise shorter in the antibiotic-exposed group than in the non-exposed group. Meta-analysis of the 10 studies revealed a statistically significant association between antibiotic use and poorer PFS, albeit with substantial heterogeneity. However, this finding was not robust: statistical significance was lost after the exclusion of seven of the ten studies in leave-one-out sensitivity analyses, and the result should therefore be interpreted with caution. Moreover, funnel plot assessments and statistical tests (Egger’s and Begg’s tests) revealed no substantial evidence of publication bias. Additional analyses of ORR and DCR demonstrated no significant differences between the two groups.

Mechanistically, ICIs work primarily by blocking inhibitory checkpoints such as CTLA-4 and PD-1/PD-L1, thereby restoring T-cell-mediated anti-tumor defenses (Ribas & Wolchok, 2018). The clinical relevance of PD-L1 in HCC has been further underscored by recent biomarker studies. For instance, Shao et al. (2023) demonstrated that the copper transporter ATP7A positively regulates PD-L1 expression in HCC cells—knockdown of ATP7A reduced PD-L1, while overexpression increased it—and that patients with ATP7A^+^/PD-L1^+^ co-expression exhibited the poorest prognosis. Accumulating evidence implicates the gut microbiota as a key modulator of ICI efficacy across cancer types, including HCC. Distinct microbial signatures appear linked to differential treatment responses. For example, in HCC, Lee et al. (2022) described a response-associated gut signature marked by enrichment of *Lachnoclostridium* and depletion of *Prevotella 9*. In another study, Zhu et al. (2024a) noted significant differences in bacterial communities between patients who did and did not benefit clinically from ICIs, whereas fungal community composition remained relatively stable. Such insights highlight the therapeutic potential of targeting the gut microbiome. In fact, interventions such as fecal microbiota transplantation have demonstrated promise in enhancing cancer immunotherapy outcomes in several studies (Duttagupta et al., 2026; Hadi et al., 2025).

The influence of the gut microbiota and its metabolites on immunotherapy responses is largely mediated through the modulation of T-cell immunity. To illustrate, Cao et al. (2025) showed that an intact gut microbiome works synergistically with ICIs to remodel the tumor immune microenvironment—specifically by boosting the proportions of CD8^+^, CD4^+^, and *γδ* T cells, dampening glycolytic metabolism, and reversing CD8^+^ T cell exhaustion into memory/effector phenotypes, thereby strengthening anti-tumor immunity. Microbial metabolites regulate T cell function, and by extension ICI efficacy, through diverse and context-dependent mechanisms. These include epigenetic remodeling, receptor signaling, metabolic reprogramming, and even acting directly as ligands for T-cell receptors (Liu et al., 2025b). For example, butyrate may enhance CD8^+^ T-cell function during anti-PD-1 therapy but potentially hinder anti-CTLA-4 therapy by inhibiting dendritic cells (Zhu et al., 2023; Coutzac et al., 2020); indole-3-propionic acid can augment anti-PD-1 responses *via* activation of the aryl hydrocarbon receptor and subsequent epigenetic changes (Jia et al., 2024); and acetic acid derived from *Bacteroides thetaiotaomicron* can promote pro-inflammatory macrophage polarization, which in turn boosts cytotoxic CD8^+^ T-cell function and suppresses HCC growth (Ma et al., 2024). Given that antibiotic treatment profoundly disrupts this delicate and functionally vital ecosystem, its capacity to influence ICI outcomes is evident. However, the effect is unlikely to be uniform, as different antibiotics perturb the microbiota in distinct ways. Therefore, future research designed to unravel the specific impacts of individual antibiotic classes or agents on ICI efficacy will be crucial for informing clinical practice.

Notably, our study preferentially adopted multivariable-adjusted effect estimates to mitigate the influence of potential confounders to the greatest extent possible, thereby evaluating antibiotic exposure as an independent risk factor. Nevertheless, the present study cannot elucidate the causal relationship or the underlying mechanisms linking antibiotic use to ICI efficacy. Numerous clinical factors influence survival outcomes in patients with malignant tumors, and residual confounding cannot be fully eliminated. In our study cohort, with the exception of a small subset of patients who received antibiotics for prophylactic purposes, the majority had already developed infectious diseases prior to antibiotic administration. Infections in advanced HCC (*e.g.*, spontaneous bacterial peritonitis, cholangitis, pneumonia) independently confer a poor prognosis and may themselves substantially affect patient survival. Consequently, it remains challenging to definitively distinguish whether mortality should be attributed to tumor progression or to concurrent infectious diseases. Future investigations should focus on patients receiving prophylactic antibiotics to further examine this issue.

Several limitations of the present study should be acknowledged. First, significant and unexplained heterogeneity was observed in the pooled analysis of PFS, which may compromise the reliability of this endpoint. Second, owing to the limited number of included studies and the insufficient granularity of the available data, we were unable to perform subgroup analyses stratified by key factors—such as specific antibiotic classes, the temporal relationship between antibiotic exposure and ICI administration, or the clinical indication for antibiotic use—which are likely to serve as important effect modifiers of the observed associations. Third, the conclusions regarding ORR and DCR are constrained by the very small number of studies reporting these endpoints. Fourth, as all included studies were retrospective in nature, the present study can only identify statistical associations between antibiotic exposure and ICI efficacy and cannot establish causality. To address these gaps and refine future clinical guidelines, large-scale, multicenter prospective studies with detailed and standardized documentation of antibiotic use are warranted.

## Conclusion

For HCC patients receiving ICI therapy, antibiotic exposure was significantly associated with reduced OS. A significant association with poorer PFS was also observed, although this finding was not robust in sensitivity analyses and should therefore be interpreted with caution. No statistically significant differences were observed for ORR or DCR.

## Supplemental Information

10.7717/peerj.21840/supp-1 Supplemental Information 1 PRISMA checklist (2020)

10.7717/peerj.21840/supp-2 Supplemental Information 2 Search strategy

10.7717/peerj.21840/supp-3 Supplemental Information 3 Further details for studies

10.7717/peerj.21840/supp-4 Supplemental Information 4 Newcastle-Ottawa scale of all study

10.7717/peerj.21840/supp-5 Supplemental Information 5 Codebook

## Additional Information and Declarations

### Data Availability

The following information was supplied regarding data availability: This is a Systematic review/Meta-analysis.
