# Supplementary material for: Association between antibiotic exposure and survival outcomes in hepatocellular carcinoma patients treated with immune checkpoint inhibitors: a systematic review and meta-analysis
Source: PeerJ. 2026 Oct 28;14:e21840. doi: 10.7717/peerj.21840 (PMC13723194; doi:10.7717/peerj.21840)
Supplement: Supplementary file 3 [file peerj-14-21840-s003.docx]

Additional File 3 Further details for studies

| Study ID | Author | immune checkpoint inhibitor type | Indications for antibiotics | Antibiotics type | Antibiotics exposure window (days relative to ICI) |
| --- | --- | --- | --- | --- | --- |
|  |  |  |  |  |  |
| 1 | Ren LY | PD-1 (camrelizumab, sintilimab, tislelizumab) | Abdominal infection (n=7), bloodstream infection (n=6), pulmonary infection (n=6), other infection (n=4), prophylactic use (n=2). (pre-PSM). | Fluoroquinolones (levofloxacin, moxifloxacin) (n=6), β-lactams (carbapenems, cefoperazone-sulbactam, ceftriaxone, piperacillin) (n=19). (pre-PSM) | within ±30 days of treatment initiation |
| 2 | Yi JZ | PD-1/PD-L1(pembrolizumab, tislelizumab, sintilimab, atezolizumab, durvalumab) | Infection: pneumonia (n=9), GI infections (n=5), UTI (n=4), skin (n=2), other (n=8); Empirical/prophylactic (n=50). | Cephalosporins (n=44), fluoroquinolones (n=20), penicillins (n=14). | within ±30 days of treatment initiation |
| 3 | Ng KYY | / | / | / | within ±30 days of treatment initiation |
| 4 | Li Y | / | Pulmonary infections (n=8), Intra-abdominal infections (n=8), Bloodstream infections (n=6), Other indications (n=7). | β-lactams (n=27), Non-β-lactams (n=2). | within ±60 days of treatment initiation |
| 5 | Pinato DJ | PD-1/PD-L1 inhibitors, CTLA-4 antagonists | / | / | within ±30 days of treatment initiation |
| 6 | Maesaka K | PD-L1 inhibitors (atezolizumab) | cholangitis (n=2), cholecystitis (n=2), hepatic abscess (n=2), pyothorax (n=1), and cystitis (n=1), as prophylaxis after transarterial chemoembolization (n=3), liver biopsy (n=2), endoscopic ultrasound-guided fine needle aspiration (n=2), endoscopic retrograde cholangiopancreatography (n=1), hepatic arterial infusion chemotherapy (n=1), hepatic encephalopathy (n=2). | cefmetazole (n=4), sulbactam/cefoperazone (n=4), cefazolin (n=2), cefepime (n=1), cefpodoxime proxetil (n=1), sulbactam/ampicillin (n=1), tazobactam/piperacillin (n=1), amoxicillin/clavulanate (n=1), levofloxacin (n=1), teicoplanin (n=1), rifaximin (n=2). | up to 30 days before treatment initiation |
| 7 | Alshammari K | PD-L1 inhibitors (Nivolumab) | Spontaneous Bacterial Peritonitis (n=6), Urinary Tract Infection (n=6), Sepsis (n=4), Upper Respiratory Tract Infections (n=2), Pneumonia (n=2). | Ceftriaxone (n=7), Ciprofloxacin (n=5), Piperacillin/tazobactam (n=3), Meropenem (n=1), Augmentin (n=2), Moxifloxacin (n=2). | up to 30 days before, during, and up to 30 days after treatment |
| 8 | Hatanaka T | PD-L1 inhibitors (atezolizumab) | a diagnosed infective disease (n=16), prophylaxis: to prevent infection due to liver tumor biopsy (n=10), treatment for infectious signs and symptoms: in case of a suspicious infective disease (n=13). | / | within ±30 days of treatment initiation |
| 9 | Wang JR | PD-1 inhibitors | / | aminoglycosides, quinolones, carbapenems, and cephalosporins. | within 30 days before and after immunotherapy |
| 10 | Fessas P | PD-1/PD-L1 inhibitors, CTLA-4 antagonists | Pneumonia (n=18), Urinary tract infections (n=17), Gastro-intestinal infections (n=17), Skin infections (n=6), Empirical-unclear source (n=112), ENT infections (n=11), Dental infections (n=6), Encephalopaty (n=14), Blood borne/septicaemia (n=3), Pre-TACE prophylaxis (n=6) | Beta-lactams (n=121), Quinolones (n=73), Macrolides (n=20), Sulfonamides (n=2), Aminoglycosides (n=1), Tetracyclines (n=8), Carbapenems (n=5), Cephalosporins (n=59), Nitroimidazole (n=15), Trimethoprim (n=6), Glycopeptides (n=16), Rifampicin (n=18) | within ±30 days of treatment initiation |
| 11 | Cheung KS | PD-1 inhibitors, CTLA-4 antagonists (nivolumab, pembrolizumab, ipilimumab) | Fever of unknown cause/empirical use (n=35), Pneumonia (n=7), Gastrointestinal bleeding (n=5), Post-TACE fever (n=5), Biliary sepsis (n=4), Ruptured HCC (n=3), Cellulitis (n=3), Urinary tract infection (n=2), Diarrhoea (n=2), Hepatic encephalopathy (n=2), Liver abscess (n=2), Spontaneous bacterial peritonitis (n=1), Thrombosed haemorrhoids (n=1), Post-SIRT fever (n=1), TURP (n=1), Salmonella bacteremia (n=1), Post-operative bile leak (n=1) | Penicillins (n=83), Cephaloporins (n=28), Macrolides (n=3), Carbapenems (n=24), Quinolones (n=26), Tetracyclines (n=2), Aminoglycosides (n=4), Nitroimidazoles (n=14), Glycopeptides (n=3), Septrin (n=3), Nitrofurantoin (n=1), Rifampicin (n=1), Rifaximin (n=2) | within ±30 days of treatment initiation |
| 12 | Spahn S | PD-L1 inhibitors (nivolumab, pembrolizumab) | / | broad spectrum cephalosporins or fluoroquinolones (n=13), rifaximin (n=8) | within ±30 days of treatment initiation |
