# Supplementary material for: Association between antibiotic exposure and survival outcomes in hepatocellular carcinoma patients treated with immune checkpoint inhibitors: a systematic review and meta-analysis
Source: PeerJ. 2026 Oct 28;14:e21840. doi: 10.7717/peerj.21840 (PMC13723194; doi:10.7717/peerj.21840)
Supplement: Supplementary file 4 [file peerj-14-21840-s004.docx]

Additional File 4 Newcastle-Ottawa Scale of all study

| No. | Study | selection | | | | comparability | outcome | | | NOS score | Average score |
| --- | --- | --- | --- | --- | --- | --- | --- | --- | --- | --- | --- |
|  |  | Cohort representativeness | Selection of the unexposed cohort | Determination of exposure factors | Free of target disease at baseline | Intergroup comparability | Determination of outcome indicators | Sufficient follow-up duration | Complete follow-up records |  |  |
| 1 | Ren LY 2026 | 1 | 1 | 1 | 1 | 2 | 1 | 1 | 1 | 9 | 8.17 |
| 2 | Yi JZ | 1 | 1 | 1 | 1 | 2 | 1 | 1 | 1 | 9 |  |
| 3 | Ng K 2025 | 1 | 1 | 1 | 1 | 1 | 1 | 1 | 1 | 8 |  |
| 4 | Li Y2025 | 1 | 1 | 1 | 1 | 2 | 1 | 0 | 1 | 8 |  |
| 5 | Pinato DJ2023 | 1 | 1 | 1 | 1 | 2 | 1 | 0 | 1 | 8 |  |
| 6 | Maesaka K 2023 | 1 | 1 | 1 | 1 | 2 | 1 | 0 | 1 | 8 |  |
| 7 | Alshammari K 2023 | 1 | 1 | 1 | 1 | 2 | 1 | 0 | 1 | 8 |  |
| 8 | Hatanaka T 2023 | 1 | 1 | 1 | 1 | 2 | 1 | 0 | 1 | 8 |  |
| 9 | Wang JR2022 | 1 | 1 | 1 | 1 | 2 | 1 | 0 | 1 | 8 |  |
| 10 | Fessas P2021 | 1 | 1 | 1 | 1 | 2 | 1 | 0 | 1 | 8 |  |
| 11 | Cheung KS2021 | 1 | 1 | 1 | 1 | 2 | 1 | 1 | 1 | 9 |  |
| 12 | Spahn S2020 | 1 | 1 | 1 | 1 | 0 | 1 | 1 | 1 | 7 |  |
