# Supplementary material for: Association between antibiotic exposure and survival outcomes in hepatocellular carcinoma patients treated with immune checkpoint inhibitors: a systematic review and meta-analysis
Source: PeerJ. 2026 Oct 28;14:e21840. doi: 10.7717/peerj.21840 (PMC13723194; doi:10.7717/peerj.21840)
Supplement: Supplementary file 5 [file peerj-14-21840-s005.docx]

**Supplementary File 5: Codebook for Categorical Variables**

This codebook provides the mapping between numeric codes and their respective categorical meanings for all variables in the dataset. All Newcastle-Ottawa Scale (NOS) items are ordinal categorical variables (scored 0, 1, or 2), where higher scores indicate better methodological quality.

| **Domain** | **Item** | **Code** | **Meaning** |
| --- | --- | --- | --- |
| **Selection** | **Cohort representativeness** | **0** | **Not representative / no description** |
|  |  | **1** | **Somewhat or truly representative** |
|  | **Selection of the unexposed cohort** | **0** | **No description / different source** |
|  |  | **1** | **Drawn from the same community** |
|  | **Determination of exposure factors** | **0** | **No description / self-report** |
|  |  | **1** | **Secure record / structured interview** |
|  | **Free of target disease at baseline** | **0** | **No** |
|  |  | **1** | **Yes** |
| **Comparability** | **Intergroup comparability** | **0** | **No adjustment for confounders** |
|  |  | **1** | **Adjusted for the most important confounder** |
|  |  | **2** | **Adjusted for >= 2 additional confounders** |
| **Outcome** | **Determination of outcome indicators** | **0** | **No description / self-report** |
|  |  | **1** | **Independent blind assessment / record linkage** |
|  | **Sufficient follow-up duration** | **0** | **Not stated / inadequate** |
|  |  | **1** | **Adequate follow-up duration** |
|  | **Complete follow-up records** | **0** | **Not stated / < 80% complete** |
|  |  | **1** | **>= 80% complete with description of losses** |
